# Blastic Plasmocytoid Dendritic Cell Neoplasm (BPDCN): Clinical Features and Histopathology with a Therapeutic Overview

**DOI:** 10.3390/hematolrep15040070

**Published:** 2023-12-08

**Authors:** Gerardo Cazzato, Marialessandra Capuzzolo, Emilio Bellitti, Giovanni De Biasi, Anna Colagrande, Katia Mangialardi, Francesco Gaudio, Giuseppe Ingravallo

**Affiliations:** 1 Section of Molecular Pathology, Department of Precision and Regenerative Medicine and Ionian Area (DiMePRe-J), University of Bari “Aldo Moro”, 70124 Bari, Italy; m.capuzzolo@studenti.uniba.it (M.C.); giovanni.debiasi@uniba.it (G.D.B.); anna.colagrande@gmail.com (A.C.); giuseppe.ingravallo@uniba.it (G.I.); 2 Anatomic Pathology Unit, “A. Perrino” Hospital, 72100 Brindisi, Italy; emilio.bellitti@asl.brindisi.it; 3 Hematology Section, Department of Emergency and Transplantation, University of Bari Medical School, 70124 Bari, Italy; katia.mangialardi@uniba.it (K.M.); fragaudio@alice.it (F.G.)

**Keywords:** BPDCN, aggressive neoplasm, hematology, PDCs, WHO, myeloid, leukemia, lymphoma

## Abstract

Blastic Plasmacytoid Dendritic Cell Neoplasms (BPDCNs) are a rare, highly aggressive hematological malignant neoplasm that primarily involve the skin, bone marrow, lymph nodes and even extra-nodal sites. The rarity and relative poor description of cases in the literature make it necessary to review and further studies that deeply investigate this entity not only in a histopathological but also molecular field. In August–September 2023, we searched MEDLINE, PubMed and Scopus for randomized controlled trials (RCTs), narrative and systematic reviews, meta-analyses, observational studies (either longitudinal or retrospective), and case series published in English in the last 25 years using the keywords BPDCN, PDCs, Blastic NK-cell lymphoma, agranular CD4+ NK leukemia/lymphoma, agranular CD4+ CD56+ hematodermic neoplasm/tumor. Despite the progress made in recent years in the diagnosis and biological understanding of the disease, until 2018 there was no clear consensus regarding its treatment and the main therapeutic schemes used were based on chemotherapy regimens already used in the treatment of lymphomas, acute lymphoblastic leukemia (ALL) and/or acute myeloid leukemia (AML). In this narrative review, we address the definition and epidemiological features of BPDCN, provide the different theories on the etiopathogenesis with particular attention to the presumed cell of origin, discuss the main clinical manifestations that provide a sign of its presence, summarize the main histopathological and immunophenotypic characteristics with special attention to the most important markers, and finally, we provide some of the most effective information on the therapeutic treatment modalities of BPDCN.

## 1. Introduction

Blastic Plasmacytoid Dendritic Cell Neoplasm (BPDCN) is a rare, highly aggressive hematological malignant neoplasm that primarily involves the skin, bone marrow, lymph nodes and even extra-nodal sites [1]. Derived from the precursors of plasmacytoid dendritic cells (PDCs, also called professional type I interferon-producing cells), it is mostly found in the elderly population (>65 years) but has also been described in other age groups [2,3] and epidemiologically, men are three times more likely to be affected than women [3]. BPDCN constitutes 0.44% of all hematological malignancies, less than 1% of acute leukemias and 0.7% of cutaneous lymphoma detected each year and corresponds to approximately 700–1000 cases estimated annually in the United States of America (USA) and Europe, respectively, while still remaining an underdiagnosed neoplasm [1,2,3,4]. It is well known that the first physician who meets a patient suffering from suspected BPDCN is the dermatologist, whose judgment and suspicion is fundamental in recognizing the possibility of the diagnosis and in reducing the time to start the therapeutic treatment which, although capable of providing an initial response, is not yet capable of significantly improving the prognosis [5,6]. Even today, the average survival is 8–14 months after the initial diagnosis. Despite the progress made in recent years in the diagnosis and biological understanding of the disease, until 2018 there was no clear consensus regarding its treatment and the main therapeutic schemes used were based on chemotherapy regimens already used in the treatment of lymphomas, acute lymphoblastic leukemia (ALL) and/or acute myeloid leukemia (AML). In this narrative review, we address the definition and epidemiological features of BPDCN, provide the different theories on the etiopathogenesis with particular attention to the presumed cell of origin, discuss the main clinical manifestations that provide a sign of its presence, summarize the main histopathological and immunophenotypic characteristics with special attention to the most important markers, and finally, we provide some of the most effective information on the therapeutic treatment modalities of BPDCN.

## 2. Materials and Methods

In August–September 2023, we searched MEDLINE, PubMed and Scopus for randomized controlled trials, narrative and systematic reviews, meta-analyses, observational studies, either longitudinal or historical, and case series published in English in the last 25 years using keywords BPDCN, PDCs, Blastic NK-cell lymphoma, agranular CD4+ NK leukemia/lymphoma, agranular CD4+ CD56+ hematodermic neoplasm/tumor.

For this narrative review, abstracts from 204 manuscripts found in the literature were assessed by two independent authors; of these, 45 were included, based on the impact of the latter studies on the history, definition, clinical features, histology and immunophenotype and treatment characteristics of BPDCN.

## 3. Results

### 3.1. Definition and Cell of Origin

BPDCN has long been disputed in an attempt to understand its actual origin and this classification uncertainty has resulted in different types of previously given nomenclatures such as Blastic NK-cell Lymphoma (obsolete), agranular CD4+ NK leukemia (also obsolete term), blastic NK leukemia/lymphoma (obsolete) and agranular CD4+ CD56+ hematodermic neoplasm/tumor [7]. In the World Health Organization (WHO) Classification of Tumors of Hematopoietic and Lymphoid Tissues 2018, IV Edition, it is stated that the cell of origin is a hematopoietic stem cell, and the normal counterpart is the precursor of PDCs. This type of dendritic cell was observed in 1958 in lymph nodes and remained shrouded in mystery for more than three decades. Although they were initially isolated in the T cell areas of human lymphoid tissue, they were soon also found in the bone marrow (BM), peripheral blood and umbilical cord, and are physiologically present also in the skin. PDCs morphologically resemble monocytes; However, classically they take on a plasma cell-like appearance, with an eccentric nucleus, and the six antigens considered markers of PDCs are CD(cluster of differentiation)123, CD303, CD304, TCL1, CD2AP and IRF8. Furthermore, they also express CD43, CD68 and Granzyme B. Their main task in the human organism is to produce type I interferon [8]. Recent gene expression profiling studies have made it possible to understand that PDCs are more closely related to the myeloid lineage rather than to the lymphoid line [7] and further data obtained from TCF4 (E2-2) ChIP-se data and gene expression changes following TCF4 knock-down revealed the similarity between normal PDCs, BPDCN cell lines and primary BPDCN, distinguishing this latter one from AML lines [7].

### 3.2. Epidemiology

From an epidemiological point of view, according to [9], BPDCN has an incidence in the USA ranging from 500 to 1000 cases per year; its global point prevalence is estimated at 12/100,000, with a higher incidence in patients aged ≥60 years and in males, and ~10% of cases occur in children, usually with a better prognosis [10,11]. It has no known racial and/or ethnic predilection [7].

### 3.3. Etiology

The mechanism of etiopathogenesis of BPDCN has not yet been clarified, although it is now clear that there is no real association with Epstein–Barr Virus (EBV) [7,12] and there may potentially be some oncogenesis pathway shared with myelodysplastic syndromes (MDS) but, currently, there is no single cytogenetic or molecular change characteristic of BPDCN.

### 3.4. Clinical Features

Clinically, the clinical suspicion of BPDCN is of paramount importance as 85% to 90% of patients present with skin lesions that can vary in shape, size and color. Classically, there are three potential presentation patterns of BPDCN: (1) one or few isolated purplish nodules (73%, the most common presentation); (2) one or few purplish bruise-like macules (12%); and (3) disseminated lesions, both macules and nodules (15%). In most cases, group 3 shows a diffuse skin disease at presentation and isolated forms tend not to evolve towards more widespread forms. The most common areas involved are the face or scalp (20%), lower limb (11%), trunk (9%), and upper limb (7%). Mucosal involvement is reported in 6%, especially in oral mucosa [13,14,15,16].

BPDCN is characterized by an aggressive behavior with rapid systemic dissemination, despite the usual indolent clinical presentation, with apparently isolated cutaneous involvement. Leukaemic dissemination might occur irrespective of the cutaneous spreading. The median bone marrow infiltration is 73%, with a fair residual bone marrow function. The most common findings in the peripheral blood are thrombocytopenia (78%), anemia (65%), and neutropenia (34%). Lymphadenopathy is identified in 56% of cases, splenomegaly in 44%. Involvement of the liver has been reported and appears to be more frequent in patients with extensive bone marrow infiltration [6,17,18,19,20]. Involvement of the tonsils, paranasal cavities, lungs, eyes and central nervous system (CNS) have also been reported [6]. A minority of cases present with leukemia without skin involvement.

The differential diagnosis includes other malignancies with cutaneous manifestations, such as CD56+ acute myeloid leukemia (AML), RUNX1-mutated AML, Nasal-type extranodal NK/T cell lymphoma, Subcutaneous panniculitis-like T cell lymphoma (SPTCL) and Cutaneous T cell lymphoma.

**Figure 1:**
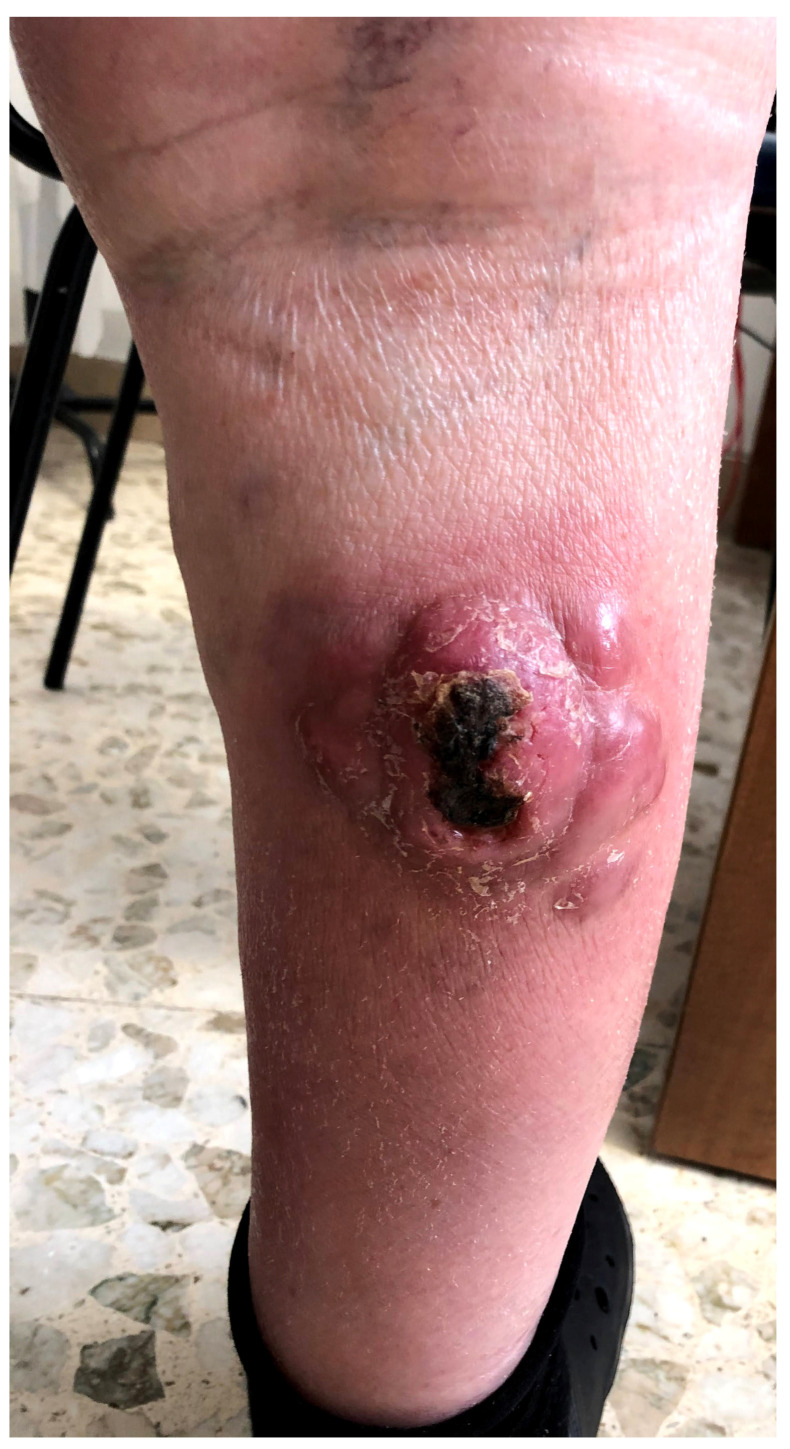
A clinical example of BPDCN in a 67-year-old male patient with an ulcerated multinodular lesion on the right leg.

Figure 1 presents a clinical example of BPDCN.

### 3.5. Diagnosis and Histopathology

Histologically, tumor cell size is consistently described as from small to medium, with either lymphoblastic or myeloblastic morphology [7]; nuclei, usually, have an irregular profile with fine chromatin and inconspicuous or prominent small nucleoli (from one to several). As for cytoplasm, it is generally agranular and not abundant, without any evidence of Auer rods [3]. The proliferation rate evaluated with Ki67+ shows a great variability, from 20% to 80%, with the presence of mitotic figures, sometimes atypical. Necrosis and angioinvasion are usually absent [7]. In this regard, Sakamoto et al. reported an “immunoblastoid” variant constituted by intermediate-sized cells including a vesicular central, open chromatin and prominent nucleoli; this different cytomorphology showed statistically significative association with MYC rearrangement [17].

#### 3.5.1. Skin

The appearance of the disease in the skin biopsy consists of a typical monomorphic and diffuse infiltrate involving the dermis, with sparing of the epidermis and the presence of a visible Grenz zone. In many cases, the involvement of the subcutaneous tissue has been described [3]; Ohgami et al. reported some patterns, such as nodular, perivascular and periadnexal [18], without involvement of adnexal structures. Necrosis is classically absent, but can be found and it is important to recognize the possibility of an angiocentric/angiotropic pattern of infiltrate.

#### 3.5.2. Lymph Node

Lymph nodes are involved in a diffuse pattern, typically in interfollicular and medullary areas [3], with the sparing of B-cell follicles.

#### 3.5.3. Bone Marrow

Bone marrow biopsies are characterized by a massive involvement or a mild and diffuse infiltration that require immunohistochemistry to highlight neoplastic cells. Hematopoietic tissue may show dysplastic features, such as trilineage dysplasia, megakaryocytic dysplasia or erythroid dysplasia.

On aspirate smears, the cells may exhibit elongated cells with irregular polarized nucleus; it can occasionally present microvacuoles in the cytoplasm and tail-shaped structure located at one edge, also called pseudopodia [3,7,19].

## 4. Immunophenotype

The immunophenotype of BPDCN includes positivity for CD4, CD45RA, CD56, CD43 and CD123, CD303, TCL1A, CD2AP, SPIB and MX1 [20,21,22], as well as the transcription factor TCF4 (E2-2), essential for driving the maturation of PDCs [23]. According to [24], approximately 8% of BPDCN cases are negative for CD4 and/or CD56, while maintaining the expression of CD123, TCL1A and/or CD303. Usually, it is possible to see the immunoexpression of CD-68 (PGM-1) in 50–80% of cases, as a dot-like expression [25], while CD7 and CD33 are commonly expressed. Some cases of BPDCN may express markers such as CD2, CD5, CD36, CD38, CD79a but are always negative for cytotoxic phenotype markers (Perforin, Granzyme B and TIA1) [26]. Other cases of BPDCN may also express antigens such as BCL6, IRF4 and BCL2 [27]. A recent paper [28] investigated in depth the immunophenotypic expression pattern in a cohort of 86 patients (109 initial patients, of which 23 were excluded due to lack of complete information) from 35 French centers, through a network of different sites. The authors reported that BPDCN cells expressed, in all cases, CD4 and CD123, together with HLA-DR and cTCL1 at a high level and very frequently expressed CD56 (99.9%), CD304 (93.6%), CD303 (75%), CD36 (92.5%), CD38 (84%), and CD45RA (89%). Furthermore, the level of CD4, CD56, and CD303 was frequently low in intensity (lower than the expression on normal T cells, NK cells, or pDCs). Regarding myeloid markers, in 47% of cases, BPDCN expressed at ≥1 marker, of which the most frequent was CD33 (43%), followed by CD117 (c-kit, 18%) and CD15 (6,4%). Of note, one case presented expressed CD13, CD33 and CD117 together to CD123, CD303 and CD304 (as previously said). Other markers such as MPO and CD14 were negative.

A total of 71.8% of cases expressed at least one T-lymphoid marker, and the most frequent was CD7 (59%) followed by CD2 (40%). CD3, CD16 and/or CD57 were never expressed. In only 15.7% of cases was at least one B-lymphoid marker expressed, the most frequent being CD22 (20%) and CD79a (7%), while CD19, CD20 and intracytoplasmic heavy-chain µ and surface immunoglobulin were always negative.

In this paper, the most frequent scenario was the co-expression of myeloid or lymphoid antigens (*n* = 30), but all combinations were possible.

Furthermore, markers such as CD11c and CD141 were not detected, with CD1c found only in 21.7% of cases at low levels.

## 5. Genetic Features

An abnormal karyotype has been frequently reported. The gross genomic imbalances present are mainly represented by the loss of genetic material and the most frequently targeted chromosomes are 5q, 12p, 13q, 6q, 15q, and 9 [6,29]. These chromosomal abnormalities are not specific and have been observed in both myeloid and lymphoid neoplasms. Molecular cytogenetic studies have identified monoallelic deletion of the NR3C1 locus at 5q31 as a recurrent abnormality in 28 percent of patients and this finding is associated with a poor clinical outcome [30,31]. Another common deletion associated with poor outcome involves the CDKN2A/CDKN2B locus at 9p21.3 [32].

### Epigenetic Abnormalities, Next-Generation Sequencing (NGS) and Whole Exome Sequencing (WES)

Some recent studies have shed light on the role of molecular biology in understanding BPDCN. In particular, in some recent papers [5,33,34] the authors analyzed mutations involving genes such as TET2, ASXL1, TP53 and NPM1, while Ceribelli M et al. [35] identified the E-box transcription factor TCF4 as a master regulator of the oncogenic activity of BPDCN, a finding demonstrated by the pro-apoptotic effect of extra-terminal domain inhibitors (BETis). Sapienza MR et al. [36], on the other hand, demonstrated that an aberration of the NF-kB pathway is involved in the molecular etiopathogenesis of BPDCN, and in another paper [3] they conducted a WES study on 14 confirmed cases, highlighting that there were mutations in genes involved in epigenetic regulation, including those implicated in DNA methylation processes (TET2 and IDH2), chromatin accessibility (ARID1a, CHD8 and SMARCA1) and histone modification such as methylation (ASXL1/SUZ12/MLL) demethylation (KDM4D), acetylation (EP300, EP400), ubiquitination (PHC1, PHC2), dephosphorylation (EYA2) and exchange (SRCAP).

## 6. Therapeutic Features

There are no formal guidelines for treatment. The therapy of BPDCN depends on several factors, such as the age, comorbidities and the previous treatments of the patient. Until 2018, in the absence of approved therapies to treat patients, regimens for acute lymphoblastic leukemia (e.g., SMILE, CALGB 9111), acute myeloid leukemia (e.g., 7 + 3) and lymphoma (e.g., HyperCVAD, ICE, CHOP) were used but with poor results [37]. In most large retrospective series (involving ≥30 patients), complete remission rates ranged from 41 to 55% and such remissions have typically had a short duration and did not translate into long-term survival benefit, with a median survival of 8 to 14 months [38]. Tagraxofusp is the first and only Food and Drug Administration (FDA)-approved treatment for BPDCN [38] for adults and children older than two years. Tagraxofusp is a fusion of a bacterial toxin with an antibody that binds to CD123, a protein expressed in BPDCN, leading to the destruction of cancer cells. In a recent clinical study involving 47 patients with BPDCN, a 90% overall response rate among previously untreated patients was observed, with the majority achieving complete remission. Serious adverse events were reported, such as capillary leak syndrome, hepatic dysfunction and thrombocytopenia, but the toxicity remains acceptable [38]. Despite normal neurological physical examination, there is a high rate of occult central nervous system (CNS) involvement in BPDCN (10% at presentation, 30% at relapse), and many regimens that have been used in BPDCN have included prophylactic intrathecal chemotherapy [39].

Allogeneic hematopoietic stem-cell transplantation (HSCT) with myeloablative conditioning for younger patients, or with reduced intensity conditioning or autologous transplantation for a selected group of older patients, has been associated with improved survival, particularly if the procedure is performed during the first complete remission. In contrast, most adults who receive no post-remission therapy relapse within two years [29]. For patients who underwent myeloablative conditioning in first CR, three-year disease-free survival (DFS) was 45% and overall survival (OS) 60%. For the entire cohort of patients, OS was 41% at three years, and no relapses were observed after 27 months past HSCT. In a univariate analysis, transplantation in first CR was associated with better outcomes, whereas age, donor, source, and the presence of chronic graft-versus-host disease had no impact on survival [40,41].

For children <2 years old there, is no defined standard of care but induction therapy with an ALL-like regimen is preferred, followed by observation rather than allogeneic HSCT in first remission. For children who achieve CR, compared with observation, it is considered that the toxicity of allogeneic HSCT in first remission outweighs the potential improvement of longer-term outcomes.

There is no consensus approach to the treatment of relapsed or refractory BPDCN. Outside of a clinical trial, treatment choice is influenced by prior therapy. For patients who were previously treated with an ALL-like regimen, tagraxofusp can be used, followed by allogeneic HSCT. In previously treated patients with relapsed/refractory disease, tagraxofusp achieved response in 60% of cases and had an 8.5-month median OS [38]. For patients previously treated with tagraxofusp, an ALL-like regimen or repeat treatment with tagraxofusp are acceptable options, followed by allogeneic HSCT.

There are also additional agents that may be useful and quite well tolerated in BPDCN. Clinical case reports have shown a response to treatment with the BCL2 inhibitor venetoclax. A phase I clinical trial is currently underway (NCT03485547); this was based on laboratory data where it was found that BPDCN cells die after treatment with Venetoclax. The investigators believe that this drug will be effective in treating patients with BPDCN [42,43]. The antifolate pralatrexate, FDA-approved for relapsed/refractory T-cell lymphomas, had efficacy in first-line and relapsed BPDCN. Furthermore, it has observed responses to enasidenib in an IDH2-mutant BPDCN [42], and to Bendamustine, which achieved a CR, and the remission was maintained for at least seven months [44].

## 7. Prognostic Features

In terms of prognosis, BPDCN is a very aggressive pathology and from the various works in the literature it can be deduced that the 5-year survival rate is <1% [41], with the vast majority of patients dying of the disease within 1 year of diagnosis. As previously mentioned, the first medical figure that a patient suffering from BPDCN generally meets is represented by the dermatologist and although it has been discussed whether patients with initial skin lesions could have a less aggressive disease course compared to patients with systemic involvement (bone, etc.) there is still no solid scientific evidence to support this [45] and HSCT absolutely represents the best candidate for increasing the survival of this type of patient [46,47]. The pediatric age would seem to represent a greater possibility of a good outcome, in terms of survival, together with an expression of TdT greater than 50% of the neoplastic cells [48], and, according to a study by Julia F. et al. [49] a greater expression of CD303 together with a higher index of neoplastic proliferation (assessed by Ki-67+) would represent a greater possibility of longer survival. On the other hand, Lucioni M. et al. reported that biallelic loss of the 9p21.3 locus would represent a negative prognostic factor [32].

## 8. Differential Diagnosis

In terms of differential diagnosis, it is very important to mention a different entity which, however, could create differential diagnosis problems for the histopathologist and which is constituted by mature plasmocytoid dendritic cell proliferation associated with myeloid neoplasm [45,50]. In patients affected by chronic myelomonocytic leukemia (CML) or myelodysplastic syndrome and/or forms of acute leukemia with monocytic differentiation, it is possible to witness the possibility of a skin infiltration of mature plasmacytoid cells, which always share the same cytogenetic anomalies with the pathology of basis, whatever it may be. Clinically, the skin lesions are not specific, and can present in the form of multiple erythematous macules, papules or even small nodules, while, from a histopathological point of view, the infiltrate is made up of mature, CD123+ PDCs, often mixed with reactive inflammatory cells [2,51,52]. The histopathological picture should not create particular problems of differential diagnosis, as the aggregates of PDCs are morphologically and phenotypically mature, with a low Ki67+ and loss of TdT immunoexpression, but, sometimes, it is possible to have diagnostic difficulties due to the fact that PDCs can simulate an inflammatory dermatitis such as cutaneous lupus erythematosus [45,52]. Table 1 summarize the most important features discussed about BPDCN.

## 9. Conclusions and Future Prospectives

BPDCN represents only 0.44% of hematological malignancies, but still has a very low survival rate, with an average survival of 8 to 14 months. In recent years, great progress has been made not only in the biological and histo-phenotypic understanding of the pathology, but also with therapeutic treatment mainly based on anti-CD123 drugs [51]. The rarity of BPDCN together with the difficulty of conducting Randomized Clinical Trials (RCTs) has certainly slowed down research in this sector of hemato-pathology, but small steps forward made in recent times give rise to hope for the immediate future. Regarding this one, a very recent position statement from North American Blastic Plasmacytoid Dendritic Cell Neoplasm Consortium [53] has interacted in a clear and exhaustive manner the main effective themes relating to the diagnostic and therapeutic aspects of BPDCN, of which we provide a brief summary here. In the first instance, a multidisciplinary approach involving a haemato-oncologist, a transplantologist, a dermatologist and close collaboration with a reference pathologist, preferably with subspecialization in dermatopathology and/or hematopathology, is fundamental. The patient with a potential and suspected diagnosis of BPDCN should undergo a complete blood count, liver and kidney function tests, and determination of serum levels of lactate dehydrogenase (LDH) and uric acid, with complete coagulation tests and a possible peripheral blood smear. Subsequently, it is necessary to perform a fine needle aspiration of the bone marrow, with flow cytometry, to determine the phenotype, as well as testing (also by immunohistochemistry) for the expression of CD123, CD4, and CD56 (mnemonic rule of “CD123-4-56”) and additional tests for markers such as TCL-1, TCF4 and CD303, which are essential for reaching the correct diagnosis (differential diagnoses already discussed previously). A Positron Emission Tomography (PET)/CT or CT scan should then be performed to study the lymph nodes and extramedullary sites, which may or may not indicate the execution of a lymph node biopsy. A complete skin examination by an expert dermatologist will then be mandatory with possible use of the Modified Severity Weighted Assessment Tool, which can be used to evaluate skin commitment as well as response to therapy [54]. A lumbar puncture (LP) with intrathecal (IT) chemotherapy should then be performed both for sampling of CSF and the potential delivery of prophylactic IT chemotherapy. Cytogenetic and NGS investigations will also be carried out for molecular mutations previously discussed and it is strictly recommended to document all visible skin lesions with faithful photographic support both at baseline and in follow-up visits.

It is essential that new clinical cases are reported in the literature to further increase knowledge of this entity and provide a greater amount of certain information capable of guiding therapeutic decisions based on Evidence-Based Medicine (EBM).

## Figures and Tables

**Table 1:** Summary of the vast majority of the features discussed in this review about BPDCN.

BPDCN	
Cell of Origin	Plasmocytoid Dendritic Cell (PDC): CD123, CD303, CD304, TCL1, CD2AP and IRF8. Furthermore, CD43, CD68 and Granzyme B
Epidemiology	incidence in the USA ranging from 500 to 1000 cases per year; prevalence 12/100,000; higher incidence in patients aged ≥60 years and in males, and ~10% of cases occur in children
Etiology	Not yet been clarified, although it is now clear that there is no real association with Epstein–Barr Virus (EBV)
Clinical Features	Solitary, localized and/or generalized plaques and nodules, sometimes with a “bruise-like” aspect
Histopathology	Monomorphous infiltrate of small/medium-sized cells in the dermis and/or subcutis with sparing of the epidermis.
Cytomorphology	Mid-sized blastoid cells, sometimes with elongated nuclei
Immumohistochemistry	Positivity for CD4, CD45RA, CD56, CD43 and CD123, CD303, TCL1A, CD2AP, SPIB and MX1 and TCF4 (E2-2)
Genetic Features	Loss of chromosomes 5q, 12p, 13q, 6q, 15q, and 9 Monoallelic deletion of the NR3C1 locus at 5q31 (worse prognosis) CDKN2A/CDKN2B locus at 9p21.3 (poor outcome)

## Data Availability

Not applicable.
